# Supplementary material for: Simultaneous Detection and Quantification of Adenine Nucleotides in Mammalian Cultured Cells by HPLC
Source: ACS Omega. 2026 Feb 26;11(9):14306–15. doi: 10.1021/acsomega.5c07459 (PMC12980428; doi:10.1021/acsomega.5c07459)
Supplement: Supplementary file 1 [file ao5c07459_si_001.pdf]

Simultaneous detection and quantification of adenine nucleotides in mammalian cultured cells by HPLC

Beatriz Kopel, Fernanda Manso Prado, Sofia Lígia Guimarães Ramos, Rafael Dias de Moura, Paolo Di Mascio, Nícolas Carlos Hoch, Marisa Helena Gennari de Medeiros, Nadja Cristhina de Souza Pinto*

Dept. de Bioquímica, Instituto de Química, Universidade de São Paulo, SP 05508-000, Brazil.

* To whom correspondence should be addressed: nadja@iq.usp.br

Keywords: adenine nucleotide quantification, RP-HPLC, ATP/ADP ratio, NAD+/NADH ratio

Table S1: ATP concentrations in µM prior to normalization.

Replicate	NT	DMSO	FK866	FCCP	02D
N1	2049,3	2568,9	2145,6	2845,4	431,4
N2	4531,7	4772,2	4653,5	4112,2	601,3
N3	1148,1	1049,3	806,9	1077,2	117,5
N4	2313,6	2361,1	1823,2	2107,3	400,5

Table S2: ADP concentrations in µM prior to normalization.

Replicate	NT	DMSO	FK866	FCCP	02D
N1	101,4	42,1	100,2	94,7	71,9
N2	106,8	26,9	56,8	182,3	87,6
N3	61,4	62,6	62,6	65,3	24,8
N4	92,3	37,5	67,1	74,1	59,9

Table S3: ADP ribose concentrations in µM prior to normalization.

Replicate	NT	DMSO	FK866	FCCP	02D
N1	11,3	3,7	2,9	7,9	9,1
N2	19,8	7,4	7,7	49,5	9,3
N3	8,5	8,9	7,1	6,7	5,2
N4	4,8	4,3	4,6	5,3	6,0

Table S4: AMP concentrations in µM prior to normalization.

Replicate	NT	DMSO	FK866	FCCP	02D
N1	7,4	6,5	11,3	5,0	23,0
N2	6,9	5,6	9,0	6,1	37,6
N3	9,9	13,8	12,7	11,0	17,2
N4	11,5	3,1	5,6	9,7	21,7

Table S5: NAD^+ concentration in µM prior to normalization.

Replicate	NT	DMSO	FK866	FCCP	02D
N1	23,9	32,4	2,4	34,8	43,0
N2	57,4	46,0	17,1	57,9	46,5
N3	15,1	15,5	4,2	19,6	9,7
N4	51,9	47,8	15,0	36,2	42,1

Table S6: NADH concentration in μM prior to normalization.

Replicate	NT	DMSO	FK866	FCCP	02D
N1	29,6	21,0	2,0	26,6	15,8
N2	47,8	32,9	34,0	56,7	54,4
N3	4,9	2,7	2,4	8,8	9,7
N4	35,9	17,4	3,8	20,0	23,3

Table S7: Analyte concentrations used in the standard curve, in μM

	ATP	ADP	ADPr	AMP	NAD+	NADH
C1	0,20	0,23	0,18	0,29	0,15	0,15
C2	0,99	1,17	0,89	1,44	0,75	0,75
C3	1,97	2,34	1,79	2,88	1,51	1,50
C4	9,86	11,71	8,94	14,41	7,54	7,52
C5	49,31	58,55	44,70	72,03	37,68	37,59
C6	197,24	234,18	178,79	288,13	150,73	150,35
C7	394,49	468,36	357,58	576,27	301,46	300,70

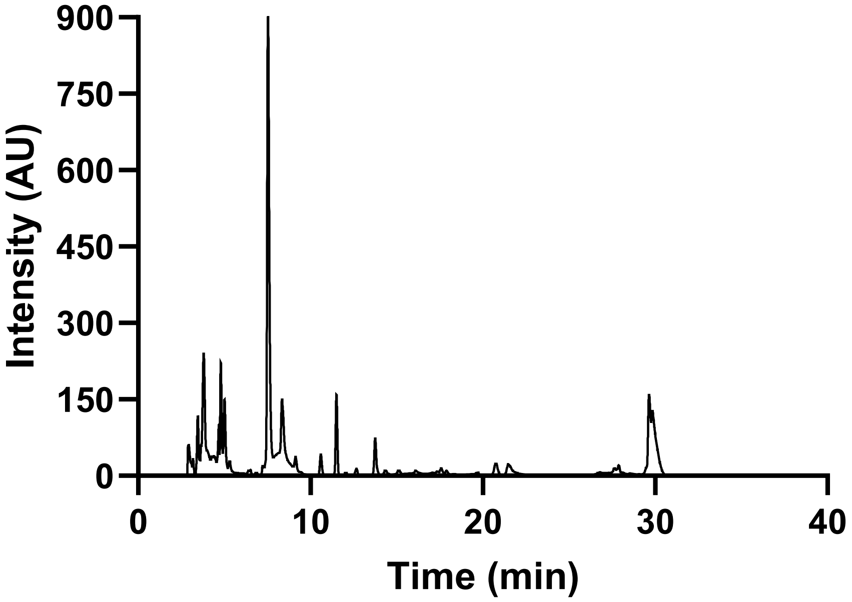

Figure S1: Representative chromatogram for the separation of an extract obtained from non-treated cells, under the same conditions described for the treated samples. The figure presents the entire chromatogram for the 40-minute running time.

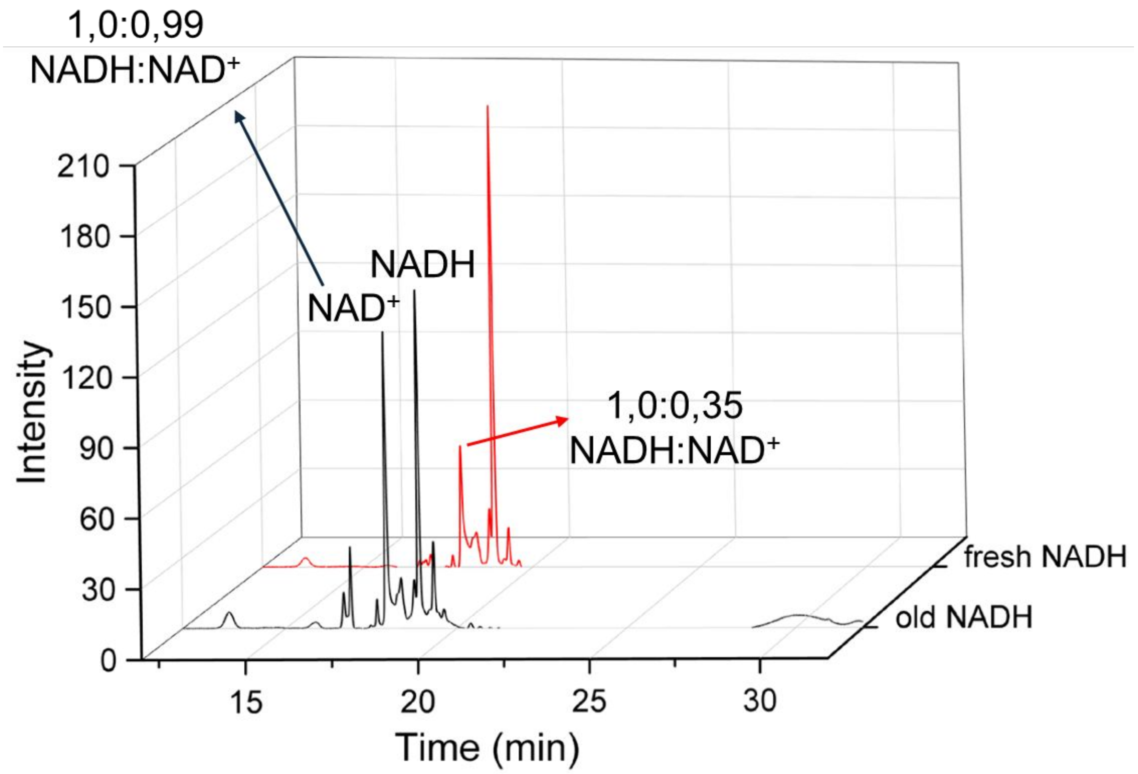

Figure S2: Degradation of NADH solutions stored at -20 ºC. Representative chromatograms of freshly made NADH standard solution (red line) and NADH standard solution of the same concentration (black line), stored for two months at -20 ºC. Both standard solutions were originally made at 50 µg/mL. Both arrows indicate the percentage of the NADH area that NAD+ peaks represent both in intact and degraded NADH standard.
